# 2215: Neuropilin-2 is expressed by activated alveolar macrophages and negatively regulates allergic airway inflammation

**DOI:** 10.1017/cts.2017.29

**Published:** 2018-05-10

**Authors:** Timothy P. Moran, Robert M. Immormino, Hideki Nakano, David Peden, Donald N. Cook

## Abstract

OBJECTIVES/SPECIFIC AIMS: Allergic asthma is a chronic lung disease driven by inappropriate inflammatory responses against inhaled allergens. Neuropilin-2 (NRP2) is a pleiotropic transmembrane receptor expressed in the lung, but its role in allergic airway inflammation is unknown. Here, we characterized NRP2 expression in lung immune cells and investigated the effects of NRP2 deficiency on airway inflammation. METHODS/STUDY POPULATION: NRP2 expression by lung immune cells from NRP2 reporter mice was determined by flow cytometry. NRP2 expression by human alveolar macrophages (AM) from healthy individuals was determined by mRNA analysis and flow cytometry. Airway inflammation in NRP2-deficient mice was assessed by bronchoalveolar lavage (BAL) cytology and inflammatory gene expression in lung tissue. RESULTS/ANTICIPATED RESULTS: NRP2 expression in lung immune cells was negligible under steady-state conditions. In contrast, inhalational exposure to lipopolysaccharide (LPS) adjuvant dramatically induced NRP2 expression in AM, as 63.3% of AM from LPS-treated mice were NRP2+ compared with 1.5% of AM from control mice. Ex vivo treatment of human AM with LPS resulted in a 1.5-fold and 2.6-fold increase in NRP2 mRNA and surface protein expression, respectively. Compared to littermate controls, NRP2-deficient mice had greater numbers of BAL leukocytes and increased lung expression of the T helper type 2 cytokines IL-4 and IL-5. Furthermore, NRP2 deficiency resulted in stochastic development of allergic airway inflammation, as spontaneous airway eosinophilia was detected in 25% (2/8) of NRP2-deficient mice compared with 0% (0/8) of littermate controls. DISCUSSION/SIGNIFICANCE OF IMPACT: NRP2 is expressed by activated human and murine AM and suppresses the spontaneous development of allergic airway inflammation. These findings suggest that NRP2 may play a key role in allergic asthma pathogenesis, and could prove to be an important therapeutic target in patients with asthma and other allergic diseases.

